# Teaching: confidence, prediction and tolerance intervals in scientific practice: a tutorial on binary variables

**DOI:** 10.1186/s12982-021-00108-1

**Published:** 2021-12-04

**Authors:** Sonja Hartnack, Malgorzata Roos

**Affiliations:** 1 Section of Epidemiology, Vetsuisse Faculty, University of Zurich, Winterthurerstr. 270, 8057 Zurich, Switzerland; 2 Epidemiology, Biostatistics and Prevention Institute, University of Zurich, Hirschengraben 84, 8001 Zurich, Switzerland

**Keywords:** Statistical interval estimates, Random sample, Bayesian analysis, Jeffreys prior

## Abstract

**Background:**

One of the emerging themes in epidemiology is the use of interval estimates. Currently, three interval estimates for confidence (CI), prediction (PI), and tolerance (TI) are at a researcher's disposal and are accessible within the open access framework in R. These three types of statistical intervals serve different purposes. Confidence intervals are designed to describe a parameter with some uncertainty due to sampling errors. Prediction intervals aim to predict future observation(s), including some uncertainty present in the actual and future samples. Tolerance intervals are constructed to capture a specified proportion of a population with a defined confidence. It is well known that interval estimates support a greater knowledge gain than point estimates. Thus, a good understanding and the use of CI, PI, and TI underlie good statistical practice. While CIs are taught in introductory statistical classes, PIs and TIs are less familiar.

**Results:**

In this paper, we provide a concise tutorial on two-sided CI, PI and TI for binary variables. This hands-on tutorial is based on our teaching materials. It contains an overview of the meaning and applicability from both a classical and a Bayesian perspective. Based on a worked-out example from veterinary medicine, we provide guidance and code that can be directly applied in R.

**Conclusions:**

This tutorial can be used by others for teaching, either in a class or for self-instruction of students and senior researchers.

**Supplementary Information:**

The online version contains supplementary material available at 10.1186/s12982-021-00108-1.

## Background

**Fig. 1:**
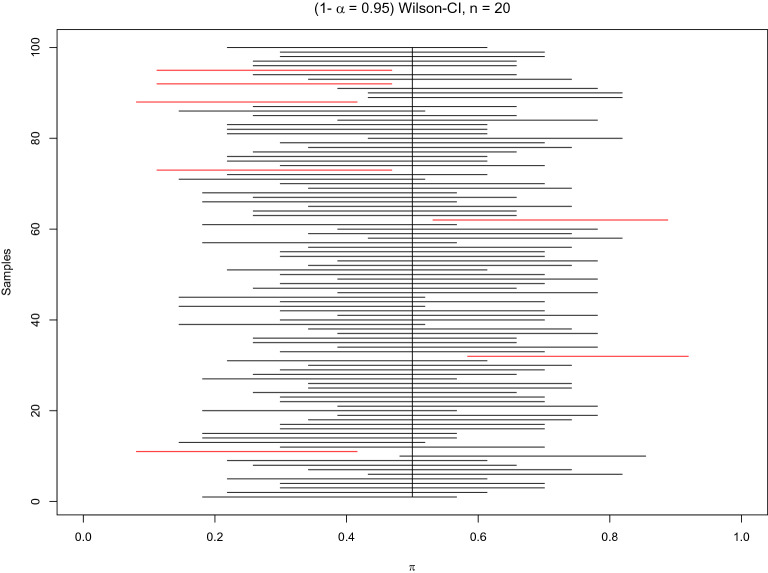
Simulation for Wilson confidence intervals. Illustration of the meaning of ($1-\alpha = 0.95$) Wilson confidence intervals CI(π) for an unknown probability π, based on 100 samples. A single sample is based on $n=20$ observations generated from a true $Be(\pi )$ distribution with $\pi =0.5$. The confidence intervals that do not cover the true probability $\pi$ are coloured red (here, 7 out of 100)

**Fig. 2:**
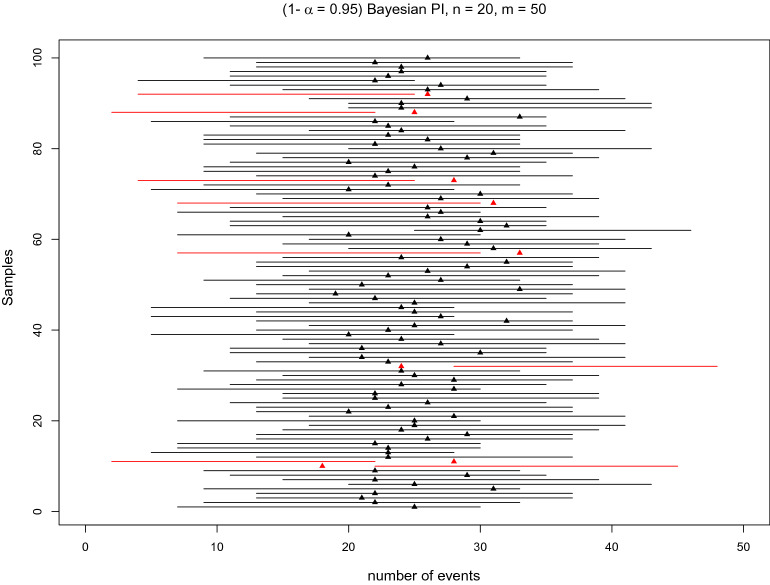
Simulation for Bayesian prediction intervals. Illustration of the meaning of $(1-\alpha = 0.95)$ prediction intervals for the number of events of interest based on 100 samples. A single sample is based on $n=20$ observations generated from a true Be(0.5) distribution, and the PI predicts the number of events in a future sample of size $m=50$. The prediction intervals, which do not cover the number of independently simulated events in $m=50$ experiments out of iid Be(0.5), are coloured red (here, 8 out of 100)

**Fig. 3:**
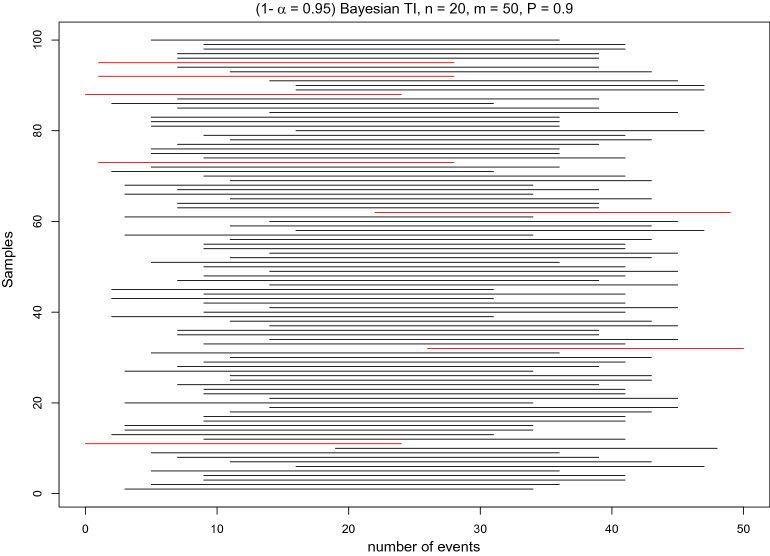
Simulation for Bayesian tolerance intervals. TIs do not need to cover any true parameter, but they contain at least a specified proportion $P$ of the population with confidence ($1-\alpha$). Illustration of the meaning of $(1-\alpha =0.95,P=0.9)$ tolerance intervals based on 100 samples. A single sample is based on $n=20$ observations generated from a true Be(0.5) distribution, and the TI predicts the number of events in a future sample of size $m=50$ specifying that at least $P=0.9$ of the results must be covered by the TI. TIs that have a content less than $P=0.9$ and do not satisfy the coverage condition ${C}_{x}\left(L,U,\theta \right)\ge 0.9$ (Additional file 1) are coloured red (here, 7 out of 100)

Statistics can be understood as a set of analytical tools to quantify uncertainty. Currently, three interval estimates for confidence (CI), prediction (PI), and tolerance (TI) are at a researcher's disposal. Confidence intervals are designed to describe a parameter with some uncertainty due to sampling errors. Prediction intervals aim to predict future observation(s), including some uncertainty present in the actual and future samples. Tolerance intervals are constructed to capture a specified proportion of a population in a future sample with a defined confidence. These intervals can be conveniently computed within the open access framework in R [1]. The main ideas behind CI, PI, and TI are presented in Figs. 1, 2 and 3. In contrast to point estimates, interval estimates consist of two numbers: lower and upper bounds. It is well known that interval estimates provide more information and support a greater knowledge gain than mere point estimates or statistical hypothesis testing. Therefore, it has been agreed that the use of interval estimates underlies good statistical practice [2]. Initiated by the British Medical Journal in 1986, other journals followed and promoted the computation of confidence intervals in their guidelines as a key pillar of journal policy [2]. Current guidelines, such as ICMJE, ARRIVE, STROBE and CONSORT suggest the usage of confidence intervals, whereas prediction or tolerance intervals are not mentioned.

The usage of CI instead of p-values was widely recommended in a current ASA statement [3], but attempts to foster PI and TI are missing. Therefore, a concise overview of the construction and interpretation of CI, PI, and TI intervals in scientific practice is urgently needed. Although we focus on an example from veterinary medicine, similar examples can be found in human and dental medicine and other areas of research. Because there are many tutorials and methodological papers for quantitative variables [4–6], we focus on binary variables.

Below, we address one part of an already published veterinary data set from Sprick et al. [7] and compute CI, PI, and TI estimates. We interpret the CI, PI, and TI results and show how the original results of Sprick et al. [7] are enhanced by new analyses. This hands-on tutorial provides functions in R and some statistical theory pertaining to both classical and Bayesian statistical frameworks.

## Main text

### Data set

The data set from Sprick et al. [7] assesses the damage inflicted by four different horseshoe materials (steel, aluminium, polyurethane, horn) on the long bones of horses. For welfare reasons, horses are increasingly kept in groups. During social interactions, kicks—particularly with the hind limbs—possibly cause fractures in the long bones, radii and tibiae when loads are applied perpendicular to the longitudinal axis. In the study from Sprick et al. [7], kicks with a comparable velocity of 16 m/s were simulated during a drop impact test setup for four different horseshoe materials. To obtain a random and representative sample, the bones were allocated to the groups to obtain a uniform distribution with respect to age, sex, and type of bone. Each group did not contain more than one bone of the same horse. We focused only on one condition: horn (2 radial or tibial fractures out of 16 kicks). The authors found a relative frequency of fractures equal to 12.5% and provided a Clopper-Pearson CI ($\pi$) (2 to 38%).

### Classical and Bayesian approaches

There are two approaches in statistics: classical and Bayesian. In the classical or frequentist branch, the unknown true parameter of interest is assumed to be fixed and can be learned or estimated by repeatedly *frequently* drawn samples of identical, independent observations from the population. Thus, classical statistics define statistical procedures by requiring certain properties to hold. Although classical statistics cover many inferential methods, the likelihood-based approaches are very popular for parametric models. By definition, the estimate of the parameter of interest is the value of the parameter for which the likelihood attains its maximum value. A 95% classical confidence interval alludes to the sampling experiment: “If one repeatedly calculates such intervals from many independent random samples, 95% of the intervals would, in the long run, correctly include the actual value of the parameter of interest” (Meeker et al. [8], p. 26).

In contrast, Bayesian methodology assumes that the parameter of interest is random, rather than a fixed quantity, and the observed sample is fixed. Bayesian procedures are valid if they are arrived at by following the Bayes theorem, which specifies how to combine a prior and the likelihood. In addition to the likelihood, containing information about unknown parameters of the data-generating model, the prior information needs to be provided. Based on the likelihood and the prior, the posterior or “post-data” [9] distribution is derived, from which Bayesian interval estimates can be read. Therefore, Bayesian interpretation describes the properties of the distribution of the true parameter after having observed the data subject to the prior. Thus, Bayesian intervals, also called credible intervals (CrI), which are based on posterior distributions, have a completely different interpretation from the repeated sampling (i.e., frequency) probabilities used in the classical statistics. The Bayesian 95% CrI contains 95% of the posterior probability of the parameter of interest.

In applications of the Bayesian methodology, the use of a minimally informative Jeffreys prior has been recommended [10]. For binary observations, the Jeffreys prior is a Beta distribution with both shape parameters $a$ and $b$ fixed at 0.5. For this particular choice of shape parameters, the prior has a minimal impact on the posterior results. In fact, for $a=b=0.5$, the sum of both shape parameters $a+b=1$ reveals that the impact of the Jeffreys prior corresponds to one observation (Additional file 1). The Jeffreys prior, which is also called the reference or default prior, is quite convenient because practitioners do not need to decide on any prior themselves.

### Random sample and point estimates

Below, we focus on one random sample with independent observations generated by a binary primary outcome at the patient, specimen or object level attaining only two values (0 = “no”, 1 = “yes”). Usually, the value 1 corresponds to an event of interest. Assume that the sample size is equal to $n$ and observations are a vector (of length $n$) of 0/1-values. From a statistical point of view, these observations are independent and identically distributed (iid) realisations of a Bernoulli distribution ($Be(\pi )$), which attains value 1 with a true probability $\pi$ and value 0 with a true probability $1-\pi$. What the researchers are interested in is the true probability $\pi$ of an event of interest. Usually, this true value $\pi$ is unknown, so experiments need to be conducted to obtain an estimate $\widehat{\pi }$ from the data that estimates the true probability $\pi$. The estimate $\widehat{\pi }$ is obtained by dividing $x$, the sum of all events of interest in the sample, by the total sample size $n$.

**Fig. 4:**
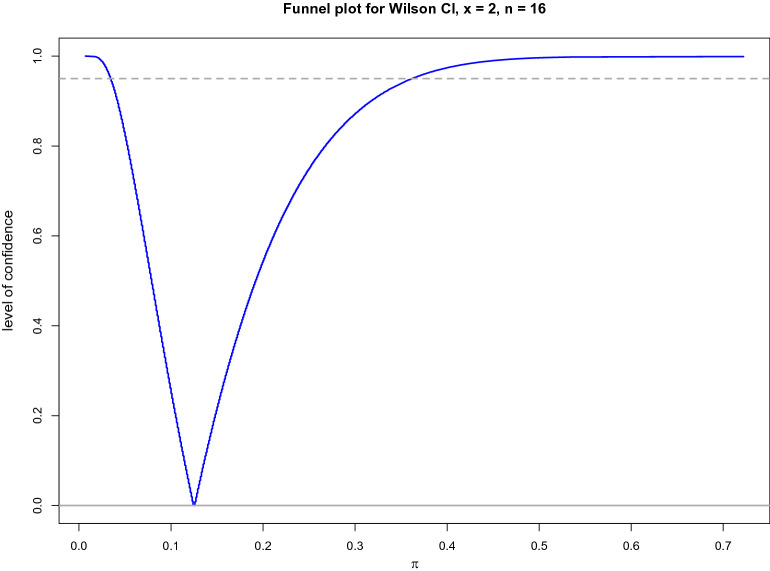
Funnel plot depicting Wilson-CIs for confidence levels ranging between 0 and 100%. The grey dashed line indicates that the Wilson 95% CI (0.034, 0.360) reported in Table 1 corresponds to the level of confidence equal to 95%. The funnel plot points at the point estimate, $\widehat{\pi }=x/n=2/16=0.125$. This indicates that one may claim that the true probability $\pi$ is equal to 0.125 with a level of confidence equal to 0

In applications, a random sample of independent 0/1 observations is usually summarised by two numbers: $n$ (sample size: the total number of considered objects in a sample) and $x$ (the number of objects that show an event of interest in the sample). For the horn data set, a total of independent $n=16$ kicks were performed, and $x=2$ of these kicks resulted in a fracture (the event of interest). These numbers are frequently presented as a relative frequency $\widehat{\pi }=x/n=2/16=0.125=12.5\%$. In statistics, the $\widehat{\pi }$ estimate is called a point estimate, which indicates what proportion of kicks resulted in a fracture when a sample size $n=16$ of independent kicks was considered. The problem with the $\widehat{\pi }$ estimate is that it is only an estimate of the true probability $\pi$ and is likely only to be close but not exactly equal to the truth. In fact, a point estimate does not show any uncertainty on its own and corresponds to confidence = 0 (Fig. 4). Therefore, to mitigate this serious drawback of point estimates, three interval estimates, CI, PI, and TI, have been developed [8]. These interval estimates share three common properties. First, they indicate an interval marked by two bounds: a lower and an upper one. Second, they require a specification of the confidence or probability level, which we set throughout at $0.95 = (1-\alpha )$ by fixing the value of the statistical error $\alpha$ at 0.05. Third, although CI, PI, and TI intervals are computed given one sample of 0/1 observations, they provide new insights into the true underlying distribution $Be(\pi )$. As we will show below, the three CI, PI, and TI interval estimates inform us about either the unknown probability $\pi$ or new realisations out of the true distribution $Be(\pi )$. In the following three subsections, we demonstrate the differences in interpretation and use of the three CI, PI, and TI interval estimates. We present either our own functions or functions implemented in specific packages in R [1].

### Applications of CI, PI, and TI

In what follows, we provide a description of methods for CI, PI, and TI combined with results, interpretation, and some remarks on their applicability. Table 1 reports CI, PI, and TI obtained for the data from Sprick et al. [7] with $x=2$ out of $n=16$ fractures with a horn impactor. Note that the interpretation of CI, PI, and TI hinges on the assumption that these data are from a random sample, i.e., long bones were collected from 16 different and unrelated animals, which are representative of the population of horses. For a binary variable, the original scale of CI (CrI) is the probability scale, and for both PI and TI, it is the count scale. Multiplication (division) of the interval bounds by the constant sample size can transform the result to the other scale and vice versa (see Table 1).

**Table 1:** Confidence interval (CI), prediction interval (PI), and tolerance interval (TI) estimates for horn: $\widehat{\pi }=x/n=2/16=0.125=12.5\%$ with confidence level $\left(1-\alpha \right)$ = 0.95, classical Wilson (W) and Bayesian Jeffreys (J) for different contents $P$, and different numbers of predicted future observations $m$

Type	$P$	$m$	Length count scale	Lower bound count ($CLB$)	Upper bound count ($CUB$)	Lower bound ($LB$) $\pi$	Upper bound ($UB$) $\pi$	Length $\pi$ scale
W-CI^c^			6^a^	0^a^	6^a^	**0.034**	**0.360**	**0.326**
J-CI^d^			6^a^	0^a^	6^a^	**0.026**	**0.344**	**0.318**
J-PI^e^		50	**19**	**0**	**19**	0^b^	0.38^b^	0.38^b^
J-PI^e^		100	**34**	**2**	**36**	0.02^b^	0.36^b^	0.34^b^
W-TI^f^	0.8	50	**22**	**0**	**22**	0^b^	0.44^b^	0.44^b^
W-TI^f^	0.8	100	**41**	**1**	**42**	0.01^b^	0.42^b^	0.41^b^
W-TI^f^	0.9	50	**24**	**0**	**24**	0^b^	0.48^b^	0.48^b^
W-TI^f^	0.9	100	**43**	**1**	**44**	0.01^b^	0.44^b^	0.43^b^
J-TI^g^	0.8	50	**22**	**0**	**22**	0^b^	0.44^b^	0.44^b^
J-TI^g^	0.8	100	**40**	**1**	**41**	0.01^b^	0.41^b^	0.40^b^
J-TI^g^	0.9	50	**23**	**0**	**23**	0^b^	0.46^b^	0.46^b^
J-TI^g^	0.9	100	**42**	**0**	**42**	0^b^	0.42^b^	0.42^b^

Original bounds are marked in bold

^a^ $CLB=n*LB$ and $CUB=n*UB$ lead to CI for the counts

^b^ $LB=CLB/m$ and $UB=UB/m$ lead to PI and TI for $\pi$

^c^W-CI: classical Wilson confidence interval

^d^J-CI: Bayesian Jeffreys credible interval

^e^J-PI: Bayesian Jeffreys prediction interval

^f^W-TI: classical Wilson tolerance interval

^g^J-TI: Bayesian Jeffreys tolerance interval

### Confidence interval (CI)

In classical statistics, the original approach to compute a CI for a mean was first described by Student [11], Neyman [12] and Welch [13]. Procedures for computation of CI for an unknown probability followed [14–16]. Morey et al. [17] and Gelman and Greenland [18] warn that classical CI can be (mis)interpreted in the Bayesian way in practice. Occasionally, users claim that there is a 95% probability that the true parameter lies between the lower and the upper bounds of the CI, although the following interpretation for a classical CI for an unknown probability $\pi$ applies: “For identical and independent repetitions of the underlying statistical sampling experiment, a $\left(1-\alpha \right)\times 100$% confidence interval will cover $\pi$ in $\left(1-\alpha \right)\times 100$% of all cases” [19].

This property of CI($\pi$) is illustrated in Fig. 1. Confidence intervals marked in red do not overlap the true probability $\pi$. Red CI($\pi$) conveys an incorrect piece of information, as the true probability $\pi$ is not included within their lower and upper bounds. Note that such an incorrect result should occur for a 95% CI ($\pi$) only in 5 out of 100 repetitions on average. In Fig. 1, there are 7 red CIs ($\pi$) out of a total of 100 simulations, resulting in an error rate of 7%.

There are several different approaches to computing CI($\pi$), such as the Clopper-Pearson CI [15], the Wilson-CI [14] and the Wald-CI [16]. Held and Sabanés Bové ([19], p. 113 – 119) show that the Wilson procedure for CI($\pi$) computation has the best statistical properties, and we recommend it for wide use in practice. The Wilson-CI($\pi$) can be conveniently computed in R using the package DescTools [20] with the command BinomCI(), specifying the number of successes $x$ out of $n$ trials. A $\left(1-\alpha \right)=95\%$ Wilson-CI is obtained by:
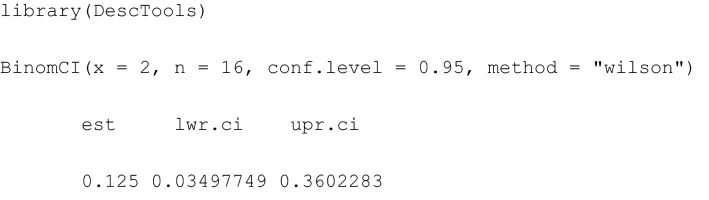


Note that there are also other packages in R offering such functionality: most prominently binom [21] with the command binom.confint() and PropCIs [22] with the command scoreci().

The interpretation of the classical Wilson-CI ($\pi$) (0.034 to 0.360) from Table 1 is as follows: For repeated, i.e., independent, identical realisations of the kick experiment with a horn impactor at a velocity of 16 m/s, the Wilson-CI($\pi$) will contain the (unknown) true probability $\pi$ of a fracture in 95% of repeated kick experiments.

### Bayesian CrI

An alternative to the classical approach is the Bayesian approach, resulting in a credible interval (CrI) based on a posterior distribution. The unknown parameter $\pi$ is contained in the $(1-\alpha )$ credible interval with probability $\left(1-\alpha \right)$.

To calculate the posterior distribution of the parameter $\pi$, the concept of conjugacy is useful. Choosing as a prior distribution, a member belonging to the same family of distributions as the posterior distribution is called a *conjugate prior* distribution [19]. For a binomial distribution, a beta distribution with a support ranging from 0 to 1 is a convenient choice for a conjugate prior [10].

**Fig. 5:**
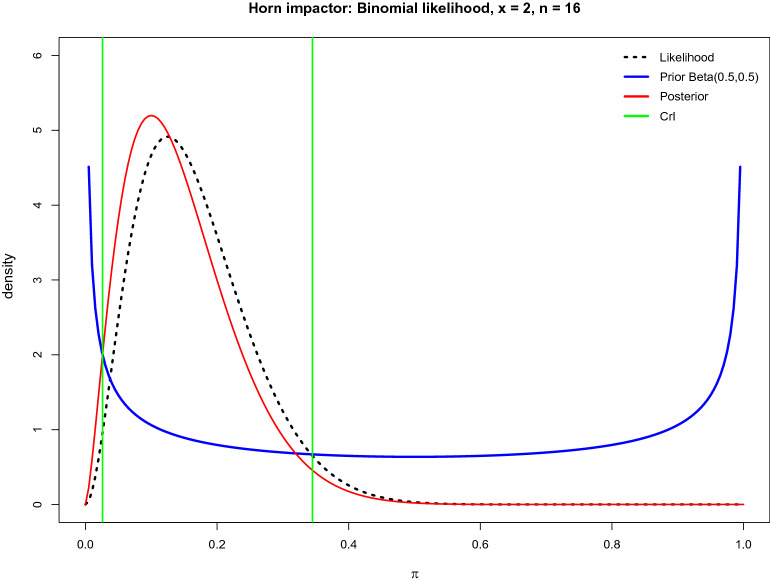
Density plots of the posterior distributions based on the Jeffreys prior (Beta(0.5,0.5)) and the binomial likelihood for $x=2$ and $n=16$ for a horn impactor from [7]. The likelihood (dotted black) and the posterior distribution (red) are similar. The $(1-\alpha =0.95)$ credible interval (0.026 to 0.344) is indicated by green lines

A Jeffreys credible interval with $x$ out of $n$ trials is computed based on a $\left(1-\alpha \right)=95\%$ probability and a minimally informative Beta prior with both parameters $a$ and $b$ fixed at 0.5 [8]. This approach is demonstrated in Fig. 5. In [16], it is proven that an equal-tailed Jeffreys CrI is always contained within the corresponding confidence interval computed according to the classical Clopper-Pearson approach and can be regarded as an improved version of the Clopper-Pearson interval. Moreover, Jeffreys CrI has good frequentist properties (coverage).

In R [1], a number of packages facilitate the calculation of Bayesian Jeffreys credible intervals, such as the package Desctools with BinomCI() [20] (see details on Jeffreys CrI in Additional file 1).
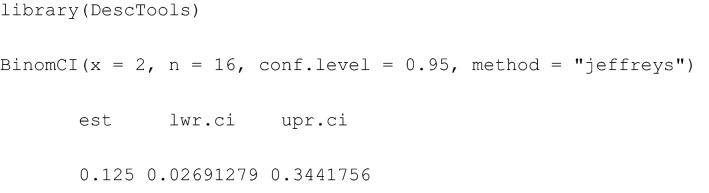


The recommended Bayesian approach leads to a Jeffreys CrI($\pi$) (0.026 to 0.344) interval estimate, shown in Table 1, and can be interpreted as follows: the posterior probability $\pi$ of a fracture in a kick experiment with a horn impactor at a velocity of 16 m/s lies in the Jeffreys interval CrI($\pi$) with probability 95%, when a minimally informative Jeffreys prior is assumed. The corresponding prior, likelihood and posterior distributions are displayed in Fig. 5.

If the main objective is the true probability $\pi$, the CI (CrI) is useful when planning the design of a new study. For example, the length of the CI (CrI) can facilitate the computation of the sample size for a future study. Given a target precision of the result (length of CI (CrI) after the study), one computes the sample size of the study necessary to achieve the required target precision of the CI (CrI).

Note that the length of both CI and CrI highly depends on the sample size $n$. The lengths of the Bayesian CrI 0.317 for 2/16 and 0.032 for 200/1600 differ drastically. This clearly demonstrates that CI ($\pi$) and CrI ($\pi )$ are mostly concerned with the value of the true probability $\pi$ but do not predict the outcome in any new future study.

### Prediction interval (PI)

The main idea behind a prediction interval is to provide an interval that covers the outcome from $m$ future observations with confidence $(1-\alpha )$, given the data ($x$ and $n$) at hand. If the main focus is on the outcome of the future $m$ observations, prediction intervals are recommended for planning future studies, power calculation, model checking or deciding whether to conduct a future trial. For details see [10, 23] and references therein.

Classical approaches to prediction intervals are mainly based on regression methods, which are conveniently applicable to quantitative primary outcomes [2]. To our knowledge, there is no simple classical procedure that shows good statistical properties in the setting with one sample and a binary primary outcome. Instead, the Bayesian methodology relying on predictive distributions is recommended in such a situation [10].

For the posterior predictive distribution, a binomial distribution is combined with a conjugate Beta prior with parameters $a$ and $b$, and the parameters of the posterior predictive distribution are determined by the sum of initially chosen $a$ and $b$ parameters and the already observed data [10]. Further details are presented in the Additional file 1. The Jeffreys PI is obtained for $a=b=0.5$. In a Bayesian approach, the unknown predicted value lies in a prediction interval with a $1-\alpha =0.95$ probability. This probability statement is induced by the posterior predictive distribution and should not be mistaken for coverage probability (see *Coverage properties and asymptotic behaviour of CI, PI, and TI*).

The following R functions compute the Bayesian Jeffreys prediction interval for the number of events of interest for a future sample of $m$ and a $(1-\alpha )$ probability level using the data from observed sample $x$ of size $n$.
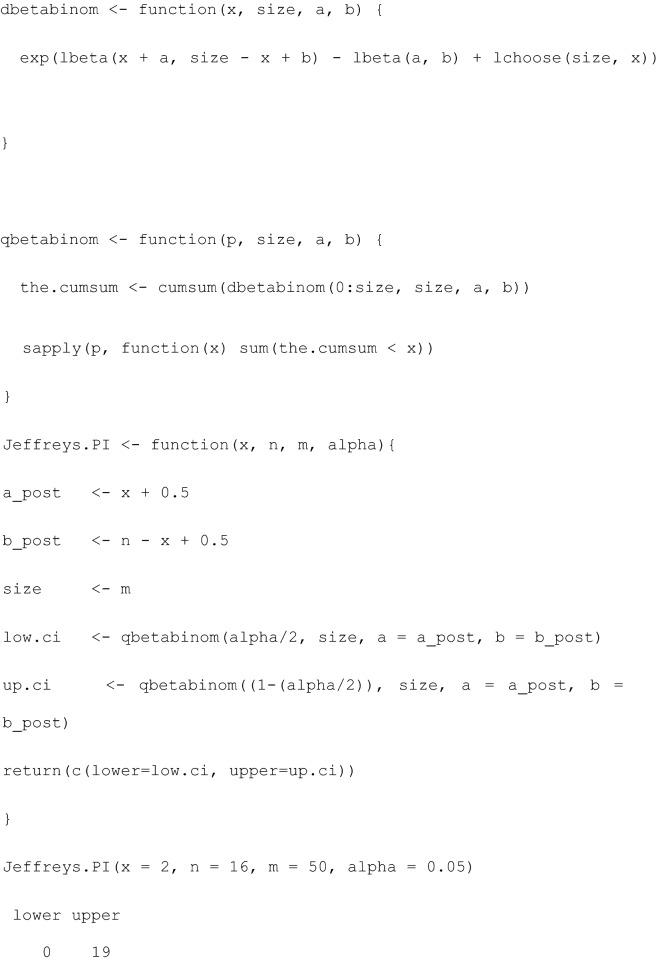


**Fig. 6:**
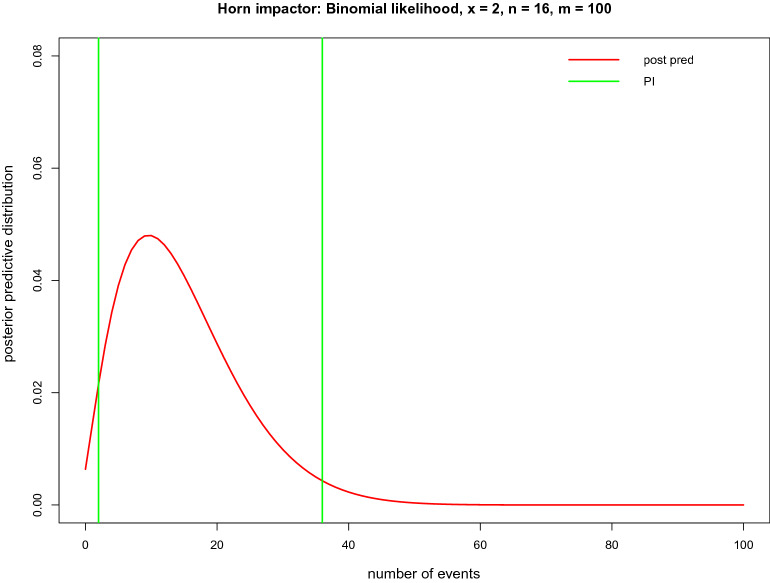
Posterior predictive distribution for a future sample of $m=100$ kicks based on the Jeffreys prior (Beta(0.5,0.5)) and the binomial likelihood for $x=2$ and $n=16$ for the horn impactor from [7]. The $(1-\alpha =0.95)$ J-PI (2 to 36) is indicated by green lines

The computation behind PI, based on the posterior predictive distribution, which predicts the number of events of interest (fractures) in a future sample of $m=100$ kicks, is illustrated in Fig. 6. Based on $x=2$ fractures in $n=16$ kicks, the Bayesian PI states that the predicted number of fractures for future experiments based on $m=50$ or $m=100$ kicks lies between (0 to 19) or (2 to 36) fractures.

A PI is less concerned with the true probability $\pi$ but rather aims to show the variability in the future data when the same experiment is conducted again several ($m$) times, given the information contained in the current ($x, n$) data. PI enables computation of lower and upper bounds on the count of observations that show the event of interest (attaining value 1) in the future sample of $m$ observations. This is shown in Fig. 2 with 100 simulations for PI with $m=50$. Red PI indicates situations when the actual observed number of events generated in $m=50$ future iid Be(0.5) experiments is not included in the PI, which predicts $m$ = 50 future observations based on $x$ and $n=20$ obtained from iid Be(0.5). The proportion of the red PI in 100 simulated PIs is 8, which is approximately equal to the assumed $1-\alpha =95\%$ confidence level. The main drawback of the PI is that it is useful to predict the performance of one, or a small number, of future observations and does not explicitly specify the proportion of the population to be covered by PI [8]. To mitigate this drawback, tolerance intervals (TIs) have been suggested.

### Tolerance interval (TI)

Frequentist definitions of tolerance intervals have a long history, dating back at least to the seminal works of Wilks [24] and Hamada et al. [25]. The origins of Bayesian tolerance intervals can be traced to Aitchison [26]. Krishnamoorthy and Mathew [27] and Meeker et al. [8] define the Bayesian tolerance interval by a frequentist formula applied to the posterior distribution. Similar to a PI, a TI enables computation of lower and upper bounds on the count of observations showing an event of interest (attaining value 1) in the future sample of $m$ observations. TI requires specification of two inputs: the percentage of the population $P$ that is covered by TI and its confidence level $\left(1-\alpha \right)$. $P$ is also called the content of the tolerance interval. For two-sided and equal-tailed tolerance intervals with an upper and a lower limit, a specified proportion $P$ of the population is contained within the bounds with a specified level of confidence $\left(1-\alpha \right)$ [27, 28] (Additional file 1). It is also possible to create one-sided tolerance intervals with respect to a threshold of interest. Both values for $\alpha$(i.e., $1-\alpha$) and $P$ can be varied independently to adjust for the requested level of confidence and the content. Several authors indicate that TIs are underused in the literature [29, 30] and are frequently not used in situations when they actually should be applied. For example, reference values for diagnostic purposes are a special case of application of tolerance values. In the R code below, $P$ denotes the chosen content or proportion of the population and does not have anything in common with p-values.

In R, the command bintol.int() available in the package tolerance [28] calculates a two-sided TI (side = 2) of content $P= 0.9$ for a future sample of size $m$ based on $x$ fractures out of $n$ kicks, based on Wilson’s approach (“WS”) together with a statistical error $\alpha =0.05$. Note that package tolerance facilitates computation of a broad range of tolerance intervals far beyond this binomial application.
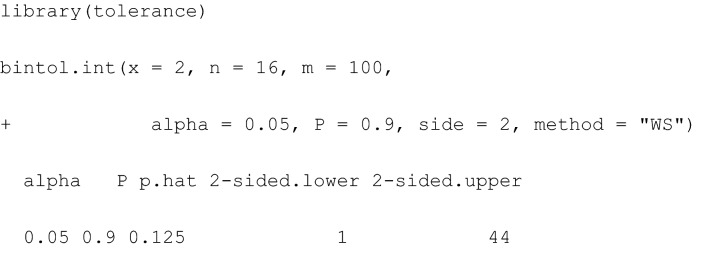


Based on current $x=2$ and $n=16$ observations, the classical Wilson-TI for the confidence level $1-\alpha =0.95$, content $P=0.8$ and a future sample of $m=50$ observations indicates a TI interval for counts (0 to 22) in Table 1. This result can be interpreted as follows: When predicting the count of radial or tibial fractures for $m=50$ future kicks, based on observed $x=2$ fractures in $n=16$ kicks, at least a proportion of 80% of future fractures (when repeating such an experiment a large number of times) will be covered by the Wilson-TI (0 to 22) interval with confidence of 95% (i.e., for repeated, i.e., independent, identical realisations of such a kick experiment with a horn impactor at a velocity of 16 m/s in 95% of repeated kick experiments). It is also possible to obtain TIs based on a Bayesian approach by specifying the method indicating that the Jeffreys approach (“JF”) is used.
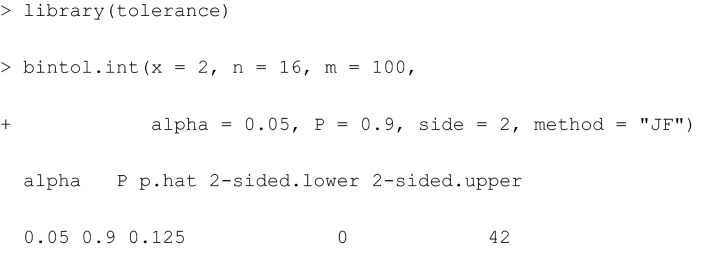


The Bayesian Jeffreys-TI for the confidence level $1-\alpha =0.95$, content $P=0.8$ and a future sample of $m=50$ observations indicates a TI interval for counts (0 to 22) in Table 1. This result can be interpreted as follows: When predicting the count of radial or tibial fractures for $m=50$ future kicks, given already observed $x=2$ fractures in $n=16$ kicks and the minimally informative Jeffreys prior, at least a proportion of 80% of future fractures (when repeating such an experiment independently a large number of times) will be covered by the Jeffreys-TI (0 to 22) interval with a probability of 95%.

Given a fixed future sample size $m$, both classical and Bayesian TI show that a larger content $P$ induces wider TI on the count scale. Moreover, for a fixed content $P$, increased future sample size $m$ is linked to narrower TI on the probability scale (Table 1).

A Bayesian TI is computed by a hybrid approach. First, a posterior distribution based on the data and a Jeffreys prior is computed. Second, the classical methodology for TI computation is applied to the posterior distribution [27]. Consequently, the interpretation of a Bayesian TI only partly benefits from the Bayesian argument. For one part of a TI, the classical “when sampling multiple times…” interpretation remains.

Figure 3 demonstrates the properties of the TI. TIs do not need to cover any true parameter $\pi$, but they contain at least a specified proportion $P$ of the population with confidence $(1-\alpha )$. Red TIs indicate TIs that are too short and do not contain the requested proportion $P$ of the population. This occurred in 7 out of 100 simulated samples.

The use of TI is recommended if a researcher wants to use the observed data to make predictions for a large number of future observations and, simultaneously, wants the interval to contain a prespecified proportion ($P$) of typical observations with confidence $\left(1-\alpha \right)$ [8]. For large sample sizes, the length of the TI approaches the quantiles of the underlying population so that the requested content $P$ is guaranteed for any future sample size $m$.

### Coverage properties and asymptotic behaviours of CI, PI, and TI

An important indicator of adequacy of interval estimates is their coverage. According to Meeker et al. ([8], p.403), the coverage probability “is the probability that the interval obtained using the procedure actually contains what it is claimed to contain, as a function of the procedure’s definition”. Coverage can be verified either by mathematical derivations or through extensive Monte Carlo simulations. The adequacy of mathematical procedures used to compute interval estimates is proven if their effective coverage levels agree well with nominal levels stipulated by assumptions imposed for their computation. For example, in the context of confidence intervals, those procedures for 95% CI computation are adequate and effectively cover the true probability $\pi$ in 95% of the cases. For CI, it was shown that not every mathematical procedure suggested for computation of 95% CI attains nominal coverage [8, 16, 19, 31–33]. For PI, the coverage was investigated by [8, 34]. Lai et al. [35] and Meeker et al. [8] present coverage for TI, called admissibility by the former. J-TI has been shown to provide a greater mean coverage probability than the nominal confidence level of 95% [8].

There were differences in the asymptotic behaviour of the three CI, PI, and TI intervals subject to increasing sample size. The length of CI converges to 0 for increasing sample size. In contrast, PI and TI stabilise at a certain stable value when the sample size is large enough with TI, resulting in longer interval estimates than PI ([8], Fig. 3.4 (binary)). Note that the discreteness of binary data leads to nonconstant coverages resembling step functions. In fact, CI, PI, and TI for a binary variable are only approximate statistical intervals [8].

### Comparison of the applicability of CI, PI, and TI

Although CI, PI, and TI share the same crucial assumption that the data at hand contain a representative random sample of kicks, the three statistical intervals address different research questions. CI (CrI) focuses on the probability parameter $\pi$ of fractures and quantifies the precision of the knowledge about this particular parameter based upon the data at hand. PI and TI do not focus on any parameter but rather predict the number of future kicks. PI and TI are calculated from the data at hand under the important assumption that the $n$ past kicks and the $m$ future kicks can be regarded as random samples from the same distribution. The PI provides information about the performance of all $m$ future kicks based upon the already observed performance of similar $n$ kicks. PIs are recommended for the prediction of a small number $m$ of future kicks (smaller than 100) ([8], p. 29). In contrast, a J-TI for $P=0.9$ and $m=50$ future kicks is not concerned with all $m=50$ future kicks but only with enclosure of a proportion $P=0.9$ of kicks. TIs apply to large numbers of future kicks $m=100$ or $m=1000$ ([8], p. 29).

Although we demonstrate the meaning of CI, PI and TI for one kick experiment, similar observations hold when the prevalence or the number of positive animals in future studies is of interest. When the focus shifts from the observed prevalence, well estimated by a CI (CrI), to the number of affected animals in a future study, PI or TI are recommended. Moreover, TIs can be used in the context of reference values for diagnostic purposes.

Thus, if we are interested in the probability of an event of interest, then we should consider a CI. If we are interested in the number of observed events in a sample of $m$ future observations, then we should consider a PI. If we are interested in the number of events in a certain proportion of observations, then we should consider a TI. In any case, a study should be carefully planned to obtain a random sample, and the decision of which interval to use should be justified depending on the objective (Meeker et al. [8], Fig. 1.1, p.16 and Table 2.1, p.24).

### Additional remarks

The meaning, properties, and applicability of confidence or credible (CI), prediction (PI) and tolerance (TI) intervals have been illustrated with a real-world veterinary data set based on a binary primary outcome. Compared with CI and PI, TI is rarely presented in teaching and publications [29, 30]. Although Meeker et al. [8] report in chapters 6, 11, 16, 18 the application of CI, PI, and TI to many case studies from different areas of research, applications of PI and TI in veterinary medicine and other areas of research seem to be scarce.

The traditional and—albeit contested by many [16]—still widely used method for obtaining confidence intervals is based on a normal approximation, the Wald CI. The Wald approach is not appropriate for very small or large proportions, i.e., when $\widehat{\pi }$ is near the boundaries of 0 or 1 and subsequently $SE\left(\widehat{\pi }\right)=\sqrt{\frac{\widehat{\pi }*(1-\widehat{\pi })}{n}}$ is close to 0. The true probability $\pi$ attains, by definition, values only in a unit (0, 1) interval. Therefore, a typical indicator of problems produced by the strongly discouraged Wald methodology is either negative lower bounds (lower than 0) or upper bounds larger than 1. If a researcher obtains such unreasonable results, she/he should be warned and should instead be encouraged to use the Wilson procedure for CI computation, as strongly recommended here.

Held and Sabanés Bové ([19], p. 113–119) show that the Wilson-CI has the best properties. They also show that although the Clopper-Pearson interval is widely used in practice, presumably due to the misleading specification “exact”, the use of this methodology is not recommended. Sprick et al. [7] used the Clopper-Pearson interval, and we have improved this analysis here by providing a Wilson-CI.

In addition to the Wilson-CI, a Bayesian alternative using Jeffreys prior is recommended. Moreover, classical and Bayesian approaches are used interchangeably. Thus, from a didactical point of view, presenting the Bayesian approach next to the classical one can help to prevent misunderstandings [30]. The usage of Bayesian intervals can be advocated on the grounds that the interpretation is more intuitive than the interpretation of classical intervals based on repeated sampling. Because there are several approaches to compute interval estimates, it is essential that the name of the effectively applied statistical methodology is clearly stated in the statistical methods section in published papers.

For prediction intervals, a Bayesian approach is recommended because in the classical context, only unsatisfactory approaches based on the Wald technique exist. Spiegelhalter et al. [10] recommend the use of PI for planning future studies. We extend this recommendation and state that both PI and TI are useful for the planning of future studies. The use of TI is similar to PI but has the advantage of explicitly specifying the content of the population covered by TI. CI can also be used for planning future studies; however, these studies should be focused on the quantification of the true parameter $\pi$.

Note that CI, PI, and TI are strongly affected by potential departures from the random sample assumption. Violation of this assumption occurs if there is any inherent structure present (e.g., clustering within animal or barn, consanguinity, members of the same household, genetic relationships, teeth in a mouth). If the assumption of a random sample is violated, statistical intervals presented in this tutorial should be used with caution. Instead, more advanced methods should be used, such as Bayesian hierarchical models ([8], chapter 17). Here, we trust that the dataset from [7] is based on long bones collected from 16 different animals. Moreover, we trust that these 16 animals are representative of the population of horses.

In the context of the so-called replication crisis, weaknesses in statistics have been described as one of the main drivers [36–38]. The usage of p-values in statistics has become highly controversial, and voices have been raised to abandon p-values in publications [39, 40]. As a solution, CIs have been proposed [41] but are also not without criticisms [18, 42]. PI and TI are less often taught and published. Therefore, in view of these ongoing discussions, a good understanding of the applicability of CI, PI, and TI can be beneficial for both researchers and scientific journals.

## Conclusion

The three types of intervals, CI (CrI), PI, and TI, serve different purposes. The decision of which interval to use should be context-driven and clearly justified. To avoid confusion and misunderstandings, all three types of intervals should be taught and presented. This hands-on tutorial on two-sided CI, PI and TI for binary variables provides guidance on applicability of these intervals from both a classical and a Bayesian perspective. A worked-out example from veterinary medicine clearly demonstrates the use of the code in R. This tutorial can be used for teaching, either in a class or for self-instruction of students and senior researchers.

## Supplementary Information

**Additional file 1.** CI, PI, and TI for a binary primary outcome.

## Data Availability

The data used are presented in the main manuscript.

## Abbreviations

CI: Confidence interval
CrI: Credible interval
PI: Prediction interval
TI: Tolerance interval
Iid: Independent and identically distributed
