# Supplementary material for: Teaching: confidence, prediction and tolerance intervals in scientific practice: a tutorial on binary variables
Source: Emerg Themes Epidemiol. 2021 Dec 4;18:17. doi: 10.1186/s12982-021-00108-1 (PMC8645111; doi:10.1186/s12982-021-00108-1)
Supplement: Supplementary file 1 — Additional file 1. CI, PI, and TI for a binary primary outcome. [file 12982_2021_108_MOESM1_ESM.pdf]

Additional file 1:
CI, PI, and TI for a binary primary outcome

for “Teaching: Confidence, prediction and tolerance intervals in scientific practice - a tutorial on binary variables” by Sonja Hartnack and Malgorzata Roos, submitted to Emerging Themes in Epidemiology

1 Theory supporting credible intervals based on Jeffreys approach

Based on the conditional probability law, the probability of Y given θ is equal to the joint probability of Y and θ divided by the probability of θ:

$$P(Y|\theta) = \frac{P(Y,\theta)}{P(\theta)} \tag{1}$$

According to Bayes rule we obtain the posterior distribution $f(\theta|y)$ given the likelihood $f(y|\theta)$ and the prior distribution $f(\theta)$:

$$f(\theta|y) = \frac{f(y|\theta)f(\theta)}{\int f(y|\theta)f(\theta)d\theta} \tag{2}$$

According to (Held and Sabanés Bové, 2014), the denominator can be written as

$$\int f(y|\theta)f(\theta)d\theta = \int f(y,\theta)d\theta = f(y) \tag{3}$$

When $f(y)$, the marginal likelihood is omitted, the posterior distribution is proportional, with proportionality constant $\frac{1}{f(y)}$, to the product of the likelihood $f(y|\theta)$ and the density of the prior distribution $f(\theta)$.
In the case of a binomial density function with

$$f(x|\theta) = \binom{n}{x}\theta^x(1-\theta)^{n-x} \tag{4}$$

and a beta prior distribution $\theta \sim Beta(a,b)$ with suitably chosen parameters $a, b > 1$,

$$f(\theta) = \frac{1}{Beta(a,b)}\theta^{a-1}(1-\theta)^{b-1} \tag{5}$$

assuming that the posterior distribution is proportional to the product of the likelihood times the prior distribution yields:

$$f(\theta|x) \propto \theta^x(1-\theta)^{n-x}\theta^{a-1}(1-\theta)^{b-1} \tag{6}$$

which can be simplified to

$$= \theta^{a+x-1}(1-\theta)^{b+n-x-1} \tag{7}$$

Thus given data x and n and the prior $Beta(a,b)$, the posterior is $Beta(a+x, b+n-x)$. It has been shown that the Jeffreys prior is "minimally informative" with respect to any other admissible priors (Bernardo, 1979; Bernardo and Smith, 2000). This means that the Jeffreys prior expresses maximal possible prior impartiality in the Bayesian setting (Gelman and Hennig, 2017) and lets the data speak for themselves. If we would assume other parameters than $a = b = 0.5$, such a prior would be less impartial than the Jeffreys prior. This means that the Uniform $U(0,1)$ prior, which is actually $Beta(1,1)$ distribution, modifies data more than the Jeffreys $Beta(0.5, 0.5)$ prior, which has a U-shaped density. Because the Jeffreys $Beta(0.5, 0.5)$ prior in our setting expresses the maximal prior impartiality, we recommend it for a wide use in applications.

Given the data x/n, which are based on the total of $x + (n-x) = n$ observations, the $Beta(a,b)$ prior is updated to the $Beta(a+x, b+n-x)$ posterior. Note that at the posterior level a and b parameters act as if the number of observations would be increased. Although there are originally $x + (n-x) = n$ observations, on the posterior there are $(a+x) + (b+n-x) = (a+b) + n$ observations. Therefore, the sum of the Beta prior parameters $a+b$ measures the impact of the prior on the data (Spiegelhalter et al., 2004). If we assume a Jeffreys $Beta(0.5, 0.5)$ prior, then its impact $a + b = 1$ is equal to one observation. Assuming a Uniform $U(0,1)$ prior which is $Beta(1,1)$ distribution would have the impact of $a+b = 2$ observations. This would also mean that the Uniform $U(0,1)$ prior would modify data more than the recommended Jeffreys $Beta(0.5, 0.5)$ prior.

2 Theory supporting prior and posterior predictive distributions

Conceptionally, the prior predictive distribution of the data corresponds to what we expect before we actually see the data. The probability distribution $f(y_n)$, with y_n being the new expected data, is a marginal probability. It is possible to obtain it by integrating out all dependence on θ of the joint probability of y_n and θ.

$$f(y_n) = \int_0^1 f(y_n, \theta) d\theta \tag{8}$$

This can be rewritten using the conditional probability law, with the probability of y given θ is equal to the joint probability of y and θ divided by the probability of θ. With

$$P(Y|\theta) = \frac{P(Y,\theta)}{P(\theta)} \tag{9}$$

the prior predictive distribution is equal to the product of the likelihood $f(y_n|\theta)$ times the prior $P(\theta)$ and integrating it out over all parameter choices.

$$f(y_n) = \int_0^1 f(y_n|\theta)f(\theta)d\theta \tag{10}$$

In the context of a binomial distribution, with conjugate beta priors, this can be expressed as

$$f(y_n) = \int_0^1 \binom{n_n}{y_n} \theta^{y_n}(1-\theta^{y_n})^{n_n-y_n} \frac{\Gamma(a+b)}{\Gamma(a)\Gamma(b)} \theta^{a-1}(1-\theta)^{b-1}d\theta \tag{11}$$

rearranged into

$$= \binom{n_n}{y_n} \frac{\Gamma(a+b)}{\Gamma(a)\Gamma(b)} \frac{\Gamma(a+y_n)\Gamma(b+n_n-y_n)}{\Gamma(a+b+n_n)} \int_0^1 \frac{\Gamma(a+b+n_n)\theta^{a+y_n-1}(1-\theta)^{b+n_n-y_n-1}}{\Gamma(a+y_n)\Gamma(b+n_n-y_n)} d\theta \tag{12}$$

and simplified to the prior predictive distribution

$$f(y_n) = \binom{n_n}{y_n} \frac{Beta(a+y_n, b+n_n-y_n)}{Beta(a,b)} \tag{13}$$

corresponding to the betabinomial distribution in (Held and Sabanés Bové, 2014, p.335).

```r
bin.pri.pred <- function(a,b,nn,yn){
# a  = shape parameter a of a beta distribution
# b  = shape parameter b of a beta distribution
# nn = sample size of new, not yet observed data
# yn = successes in new, not yet observed data
  return(exp(log(choose(nn, yn))+log(beta(a+yn,b+nn-yn))-log(beta(a,b))))
}
```

Conceptionally, once data have been observed, the posterior predictive distribution can be calculated. The question is, what value of new data y_n we would expect to obtain if the experiment would be repeated after old data y_o from a previous experiment have already been observed. Thus, with $f(y_n|y_o)$ being considered as a marginal probability which we could get from integrating out the joint probability of y_n and θ:

$$f(y_n|y_o) = \int_0^1 f(y_n, \theta|y_o)d\theta \tag{14}$$

Based on Bayes rule of conditional probability, this can be rewritten as

$$\int_0^1 f(y_n|\theta, y_o) \cdot f(\theta|y_o)d\theta \tag{15}$$

Regarding the first factor of $f(y_n|\theta, y_o)$, when we condition on θ, the new observation y_n is independent of y_o. Therefore,

$$\int_0^1 f(y_n|\theta) \cdot f(\theta|y_o) d\theta \tag{16}$$

The second factor, corresponds to the posterior distribution from the previous experiment. The first factor, $f(y_n|\theta)$ is the likelihood. After integrating out over all parameter choices we can obtain the posterior predictive distribution. When implementing this into R, the trick consists of updating the initially chosen a and b with the already observed data (y_o, n_o).

$$a = a_o + y_o \tag{17}$$

and

$$b = b_o + n_o - y_o \tag{18}$$

In the context of a binomial distribution, the posterior predictive distribution can be expressed as

$$f(y_n|y_o) = \binom{n_n}{y_n} \frac{\Gamma(a_o + b_o + n_o)}{\Gamma(a_o + y_o)(b_o + n_o - y_o)} \frac{\Gamma(a_o + y_o + y_n)\Gamma(b_o + n_o - y_o + n_n - y_n)}{\Gamma(a_o + b_o + n_o + n_n)} \tag{19}$$

and simplified into

$$f(y_n|y_o) = \binom{n_n}{y_n} \frac{Beta(a_o + y_o + y_n, b_o + n_o - y_o + n_n - y_n)}{Beta(a_o + y_o, b_o + n_o - y_o)} \tag{20}$$

representing an updated version of the betabinomial distribution in (Held and Sabanés Bové, 2014, p.335).

```
bin.post.pred <- function(a,b,nn,yn,no,yo){
# a  = shape parameter a of a beta distribution
# b  = shape parameter b of a beta distribution
# nn = sample size of new, not yet observed data
# yn = successes in new, not yet observed data
# no = sample size of old, already observed data
# yo = successes in old, already observed data
return(exp(log(choose(nn, yn))
           +log(beta(a+yo+yn,b+no-yo+nn-yn))
           -log(beta(a+yo,b+no-yo))))
}
```

Jeffreys Bayesian method for binomial prediction intervals
Jeffreys method can be viewed as a Bayesian binomial predictive distribution (Meeker et al., 2017), based on quantiles of the beta-binomial distribution considered as a Bayesian predictive distribution. It is an extension of the construction of a confidence interval, including also m, the size of the trials in a future Bernoulli experiment. Here the sample size parameter is n, the shape parameters a and b are set to 0.5 in Jeffreys approach. Similar to the posterior predictive distribution described above, the prior information is updated by the already observed data.

$$P[qbetabinom(\frac{\alpha}{2};m;x+a,n-x+b),qbetabinom(1-\frac{\alpha}{2};m;x+a,n-x+b)] = 1-\alpha \tag{21}$$

3 Tolerance intervals

According to Young (2016) statistical tolerance intervals (TI) of the form $(1-\alpha, P)$ provide bounds to capture at least a specified content proportion P of the samples population with a given confidence level $(1-\alpha)$. P is also called the *content* of the tolerance interval, and $(1-\alpha)$ reflects the sampling variability in this classical approach. It is possible to construct two-sided tolerance intervals, bounded by an upper and a lower limit. In between these, a specified content proportion is supposed to lie with a specified level of confidence. It is also possible to create one-sided tolerance intervals, with one single bound above or below a specified content proportion is found with a defined confidence level.
According to Young (2010), for a random variable X with a cumulative distribution function $F_X(.;\theta)$ and θ a vector of parameters characterizing the distribution, then

$$C_X(L,U,\theta) = F_X(U;\theta) - F_X(L;\theta) \tag{22}$$

C_X is the coverage, i.e. with confidence $(1-\alpha)$ of the two-sided interval with the lower bound L and the upper bound U. Thus,

$$P\big[C_X(L,U,\theta) \geq P\big] \geq 1-\alpha \tag{23}$$

3.1 References

Bernardo, J., 1979. Reference posterior distribution for Bayesian inference. J. R. Stat. Soc. Ser. B 41, 113-147

Bernardo, J., Smith, A. 2000. Bayesian Theory. John Wiley Sons, Ltd.

Gelman, A., Hennig, C., 2017. Beyond subjective and objective in statistics. J. R. Stat. Soc. Ser. A 180, 967-1033.

Held, L., Sabanés Bové, D., 2020. Likelihood and Bayesian Inference. 2nd ed. Heidelberg: Springer-Verlag Berlin Heidelberg.

Meeker, W., Hahn, G.J., Escobar, L., 2017. Statistical Intervals. A guide for practitioners and researchers. Wiley, New Jersey.

Spiegelhalter, D.J., Abrams, K.R., Myles, J.P., 2004. Bayesian Approaches to Clinical Trials and Health-Care Evaluation. John Wiley and Sons, Chichester.

Young, D.S., 2010. tolerance: An R Package for Estimating Tolerance Intervals. J. Stat. Softw. 36:1-39.

Young, D., 2016. Normal tolerance interval procedures in the tolerance package. The R Journal, 8, 2.
